# Infrared, Raman and Magnetic Resonance Spectroscopic Study of SiO_2_:C Nanopowders

**DOI:** 10.1186/s11671-017-2057-1

**Published:** 2017-04-24

**Authors:** Dariya Savchenko, Vladimir Vorliček, Ekaterina Kalabukhova, Aleksandr Sitnikov, Andrii Vasin, Dmytro Kysil, Stanislav Sevostianov, Valentyn Tertykh, Alexei Nazarov

**Affiliations:** 1 National Technical University of Ukraine “Igor Sikorsky Kyiv Polytechnic Institute”, pr. Peremohy 37, Kyiv, 03056 Ukraine; 2 Institute of Physics of the Czech Academy of Sciences, Na Slovance 2, Prague, 182 21 Czech Republic; 3 V.E. Lashkaryov Institute of Semiconductor Physics, NAS of Ukraine, pr. Nauky 41, Kyiv, 03028 Ukraine; 4 O.O. Chuiko Institute of Surface Chemistry, NAS of Ukraine, 17, General Naumov str, Kyiv, 03164 Ukraine

**Keywords:** EPR, Raman spectroscopy, Carbosil, Carbon-related defects, Carbon nanodots

## Abstract

Optical and magnetic properties of SiO_2_:C nanopowders obtained by chemical and thermal modification of fumed silica were studied by Fourier transform infrared spectroscopy, Raman, continuous wave (CW) electron paramagnetic resonance (EPR), echo-detected EPR and pulsed electron nuclear double resonance (ENDOR) spectroscopy. Two overlapping signals of Lorentzian lineshape were detected in CW EPR spectra of the initial SiO_2_:C. The EPR signal at *g* = 2.0055(3) is due to the silicon dangling bonds, which vanishes after thermal annealing, and the second EPR signal at *g* = 2.0033(3) was attributed to the carbon-related defect (CRD). The annealing of the SiO_2_:C samples gives rise to the increase of the CRD spin density and shift to the higher *g*-values due to the appearance of the oxygen in the vicinity of the CRD. Based on the temperature-dependent behavior of the CRD EPR signal intensity, linewidth and resonance field position we have attributed it to the spin system with non-localized electrons hopping between neighboring carbon dangling bonds, which undergo a strong exchange interaction with a localized spin system of carbon nanodots. The observed motional narrowing of the CRD EPR signal in the temperature interval from 4 to 20 K indicates that electrons are mobile at 4 K which can be explained by a quantum character of the conductivity in the vicinity of the carbon layer. The electrons trapped in quantum wells move from one carbon nanodot to another by hopping process through the energy barrier. The fact that echo-detected EPR signal at *g* = 2.0035(3) was observed in SiO_2_:C sample annealed at *T*_ann_ ≥ 700 °C serves as evidence that non-localized electrons coexist with localized electrons that have the superhyperfine interaction with surrounding ^13^C and ^29^Si nuclei located at the SiO_2_:C interface. The presence of the superhyperfine interaction of CRD with ^1^H nuclei indicates the existence of hydrogenated regions in SiO_2_:C sample.

## Background

Silica-carbon SiO_2_:C composites (carbosils) owing to their high electrical conductivity, high thermal stability and high resistance to organic solvents are widely used in industry as adsorbents for medicinal, analytical and extraction purposes. In addition, the nanostructured SiO_2_:C powders are considered as a prospective luminescent material which can be used for the conversion of ultraviolet radiation into the white light emission in gas-discharge light source and light diodes [1].

However, the nature of the broadband photoluminescence (PL) of carbon incorporated nanostructured silica remains unclear. At the same time, it was recently demonstrated that carbon nanodots, including the graphene nanodots and graphene oxide nanodots, exhibit a broadband PL in the near ultraviolet and visible spectral ranges [2–5]. The red spectral shift of the PL observed in annealed SiO_2_:C nanopowders and porous SiO_2_:C layers was attributed to an increase of the size of carbon clusters [6].

Therefore, the analysis of the carbon phases and carbon local electronic structure is of great importance to improve the properties of the SiO_2_:C materials and to find out the relation between PL and carbon phase incorporated in the SiO_2_ nanostructured matrix.

The electron paramagnetic resonance (EPR) is an excellent method to study the carbon local electronic structure. EPR has been proven to be a very powerful technique to study the different forms of carbon, including hydrogenated [7, 8] and hydrogen-free amorphous carbon [9], amorphous silicon-carbon [10], nanoporous carbon [11] and coals [12, 13]. All of these materials usually contain unpaired electrons, which serve as a basis for EPR investigations. The carbon-related paramagnetic centers have been studied also in carbosils [14] and SiO_2_:C based composites like oxidized mesoporous carbon-silica nanocomposite [15], carbon-silica sol-gel prepared nanopowders [16] and carbogenic nanodots [17].

Recently we have published the results of EPR studies of the fumed silica carbonized by means of pyrolysis of CH_2_Cl_2_, subjected to the oxidation and passivation treatment [18]. It was found that the EPR spectrum of initial, oxidized and passivated SiO_2_:C nanopowders consists of a superposition of two EPR signals with different linewidth and almost coinciding *g*-factors at *g* = 2.0033 which were attributed to the carbon-related defects (CRD) with *S* = 1/2. The existence of the narrow (~0.2 mT) and broad (~1.2 mT) EPR signals was explained by the presence of the carbon clusters of different sizes coupled with each other by the spin exchange interaction. It was found that the oxidation and passivation treatment of SiO_2_:C lead to the increase of the spin concentration of the paramagnetic centers. Based on the temperature-dependent behavior of the EPR signal intensity, linewidth and resonance field position the CRD was attributed to the spin system with non-localized electrons, which are in a strong coupling with a localized spin system. The motional narrowing of the EPR linewidth was well described by a variable range hopping law at low temperatures and the oscillating character observed for the temperature dependence of the CRD signal intensity at *T* = 200–440 K was explained by the presence of the carbon nanodots at the SiO_2_:C surface and quantum character of the carbon layer conductivity.

In this work the optical and magnetic properties of the carbonized silica (SiO_2_:C) nanocomposites obtained by chemical modification of fumed silica were studied by Fourier transform infrared spectroscopy (FTIR), Raman, continuous wave (CW) electron paramagnetic resonance (EPR), echo-detected EPR (ED EPR), and pulsed electron nuclear double resonance (ENDOR) spectroscopy in the temperature interval from 4.2 to 292 K. It was found that some magnetic properties in SiO_2_:C nanocomposites prepared by chemical modification of fumed silica are similar to those previously obtained in SiO_2_:C nanocomposites prepared by means of pyrolysis of CH_2_Cl_2_. Both types of SiO_2_:C nanocomposites revealed the EPR signal from CRD with non-localized electrons hopping between carbon nanodots on the surface of the of SiO_2_ nanoparticles. The significant increase of the spin density of the non-localized electrons was observed in both types of SiO_2_:C nanocomposites after chemical and annealing treatment. However, the annealing treatment of the SiO_2_:C nanocomposites prepared by chemical modification of fumed silica gives rise to the appearance of the oxygen in the vicinity of the CRD. In addition, it was found that in the SiO_2_:C nanocomposites annealed at *T*_ann_ ≥ 700 °C the non-localized electrons coexist with localized electrons that have a superhyperfine interaction with surrounding ^13^C and ^29^Si nuclei located at the SiO_2_:C interface. The analysis of the EPR signal linewidth revealed that the spin-lattice relaxation time of the CRD is shorter in the SiO_2_:C sample annealed at 700 °C than that in SiO_2_:C sample annealed at 800 °C. This fact indicates that the structural environment of the CRD in those two samples is different. The significant decrease of the PL intensity observed in SiO_2_:C nanocomposites annealed at 800 °C also suggests the reconstruction of the nanostructured carbon layer. The presence of the superhyperfine interaction of CRD with ^1^H nuclei indicates the existence of hydrogenated regions in SiO_2_:C samples. Thus, the CW EPR in combination with pulse EPR methods allowed us to get the detailed information about carbon phases and carbon local electronic structure incorporated in the SiO_2_ nanostructured matrix as a function of annealing temperature.

## Methods

The pristine fumed silica (aerosil A300) with specific surface area of 300 m^2^/g and 10–12 nm particle size was treated with a toluene solution of phenyltrimethoxysilane (PTMS) (C_9_H_14_O_3_Si). Then, the (H_3_CO)_3_SiC_6_H_5_ molecules were grafted to the surface via reaction described by the following scheme [19]:

$$ \equiv \mathrm{SiOH}\kern0.37em +\kern0.37em {\left({\mathrm{H}}_3\mathrm{CO}\right)}_3\mathrm{S}\mathrm{i}{\mathrm{C}}_6{\mathrm{H}}_5\to \equiv \mathrm{SiOSi}{\left(\mathrm{OC}{\mathrm{H}}_3\right)}_2{\mathrm{C}}_6{\mathrm{H}}_5+\kern0.37em \mathrm{C}{\mathrm{H}}_3\mathrm{O}\mathrm{H} $$

Thus, after the chemical treatment, we have obtained the silica nanopowder with –Si(OCH_3_)_2_C_6_H_5_ groups attached to the surface of the SiO_2_ nanoparticles, i.e., SiO_2_ : (OCH_3_)_2_C_6_H_5_. Afterwards, the samples were annealed at temperatures *T*_ann_ = 500–800 °C in N_2_ flow at atmospheric pressure for 30 min. The thermal annealing resulted in the gradual effusion of hydrogen and part of hydrocarbon radicals from the material due to thermal decomposition of (OCH_3_)_2_C_6_H_5_ groups. The decomposition of hydrocarbons inside the material and carbon precipitation after annealing at higher temperatures results in the formation of nanocomposite SiO_2_:C powder. It was found that the samples changed their color under annealing: the initial SiO_2_:C sample has a white color, while the SiO_2_:C sample annealed at 500 °C was light-brown and the SiO_2_:C samples annealed at 700 and 800 °C were dark-brown and black, respectively.

Local interatomic bonding was characterized by FTIR in transmission mode using Nicolet 6700/Nicolet Continuum (Thermo Fisher Scientific) equipped with IR/VIS-microscope that allows measuring IR-transmission from 50 × 50 μm selected area of the powder sample without using dispersion in KBr pellet. This method is very efficient for highly dispersive and IR-transparent powders. The SiO_2_:C samples annealed at 800 °C exhibited pure IR-transparency: therefore, it was examined by common KBr pellet sampling procedure.

The Raman measurements were performed in the standard backscattering geometry. Especially, the 514.5 nm (Ar-ion laser) and 633 nm (He-Ne laser) excitations with Renishaw Ramascope, Model 1000 and 785 nm (diode laser) excitation with the Renishaw inViaReflex spectrometer were used. Prior to the Raman measurements, the sensitivity of the samples to the illumination was checked and no changes (either bleaching or darkening) were observed. The samples annealed at 500–700 °C showed rather intensive PL decaying with the increasing time of illumination; therefore, the Raman spectra were recorded when some approximately “saturated” state was achieved.

The CW and pulsed EPR measurements were performed on X-band (9.4–9.7 GHz) Bruker ELEXYS E580 spectrometer in the temperature range from 130 to 10 K. The CW EPR experiments were carried out using the ER 4122 SHQE SuperX High-Q cavity, while for the pulsed EPR and ENDOR measurements the EN 4118X-MD5 and EN 4118X-MD4 cavities were used, respectively. The ED EPR spectra were measured using two-pulse Hahn echo sequence: *π*/2 – *τ* – *π* – *τ* – echo. The pulsed Mims ENDOR spectra were recorded with different pulse delays *τ* between the first two *π*/2 microwave pulses and subsequently added to minimize the suppression effects.

## Results and Discussion

### FTIR Spectroscopy Results

**Fig. 1:**
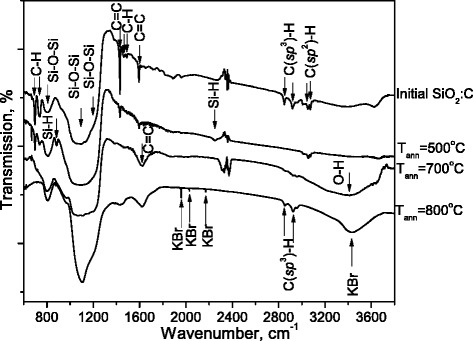
The FTIR spectra of the initial and annealed SiO_2_:C samples

FTIR spectra measured in the initial and annealed SiO_2_:C samples are presented in Fig. 1. In the initial SiO_2_:C samples, the IR-bands associated with C-H stretching (C(*sp* ^3^)-H_n_: 2800–3000 cm^−1^, C(*sp* ^2^)-H_n_: 3000–3100 cm^−1^), C = C stretching in benzene rings (narrow bands at 1593 cm^−1^ and 1427 cm^−1^), “benzene fingers” due to overtone/combination vibrations in benzene rings (at 1700–2000 cm-1) and C-H bending vibration modes (690, 730, 1470, and 1490 cm^−1^) were observed.

After sample annealing at 500 °C the FTIR spectra revealed a significant reduction of the absorption bands associated with benzene rings and almost complete vanishing of C-H bands (C(*sp* ^3^)-H_n_: 2800–3000 cm^−1^). No signs of benzene rings were observed in IR-spectra after sample annealing at 700 °C. Instead of a narrow band at 1593 cm^−1^, one can see now the broad band centered at about 1613 cm^−1^ that is a manifestation of disordered C = C bonds in amorphous carbon precipitates. Taking into account a well defined O-H stretching band in the range of 3000–3700 cm^−1^ one may expect a contribution of the O-H bending band (1630 cm^−1^) to absorption in the range of 1540–1670 cm^−1^. It is worth noting that weak, but the well-detectable band at 3000–3100 cm^−1^, indicates a presence of carbon-hydrogen bonds (C(*sp* ^2^)-H).

The increase of annealing temperature up to 800 °C results in a strong reduction of transparency of the material, presumably due to a strong increase of free electron concentration in graphite-like precipitates. It is approved by a strong increase of relative intensity of the C = C band at 1590 cm^−1^. The IR spectra of SiO_2_:C samples annealed at 800 °C exhibit relatively strong C-H band at 2800–3000 cm^−1^ (stretching C(*sp* ^3^)-H) and 1470 cm^−1^ (C-H bending). In addition, there is a well defined shoulder at 1700 cm^−1^ that is obviously due to carbonyl groups C = O.

### Raman Spectroscopy Results

**Fig. 2:**
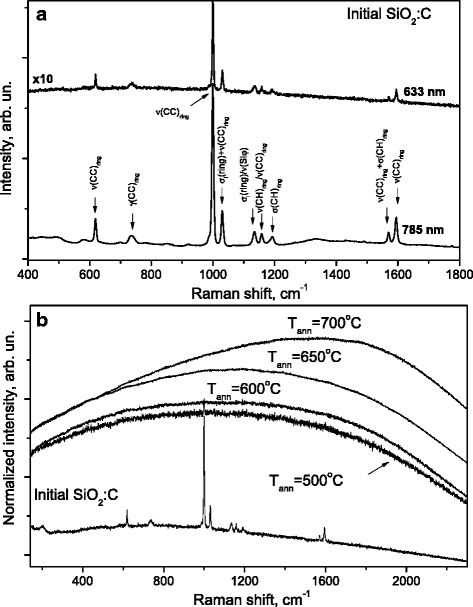
The Raman spectra of the initial SiO_2_:C sample measured with different laser wavelengths (**a**) and the comparison of Raman spectra measured in the initial and annealed SiO_2_:C samples with 633 nm excitation (**b**)

Raman scattering measurements under UV and visible excitation were strongly affected by luminescence background caused by laser probe radiation. With the aim to minimize the PL background, the Raman measurements were performed using red and infrared excitation. Figure 2a shows the Raman spectra of the initial SiO_2_:C sample obtained with red (633 nm) and IR (785 nm) excitations. The observed sharp Raman peaks are superimposed on the weak PL background. There is an excellent agreement with the Raman positions (frequencies) of the features observed with both excitations. It means that they are really the Raman features. In accordance with [20], the observed peaks were assigned to PTMS.

The Raman spectra of initial and annealed SiO_2_:C samples are compared in Fig. 2b. The temperature treatment of the SiO_2_:C powders results in the strong rise of the PL intensity and suppression of the Raman features. Just a small residue of the dominating feature at 1000 cm^−1^ is preserved in the sample annealed at 500 °C. A shift of the PL maximum towards the higher wavenumbers (relative), i.e., to longer wavelengths (on the absolute scale) is observed and it appears to agree with the results of [21]. As the PL intensity increase with the increasing of the treatment temperature is rather dramatic, the Raman spectra intensity was normalized to its maximum value to make the spectra easily comparable. We did not observe any feature in annealed samples, which could be identified with the Raman spectrum of SiO_2_. It can be explained by the fact that SiO_2_ nanoparticles are completely coated by PMTS and the exciting light does not reach SiO_2_.

**Fig. 3:**
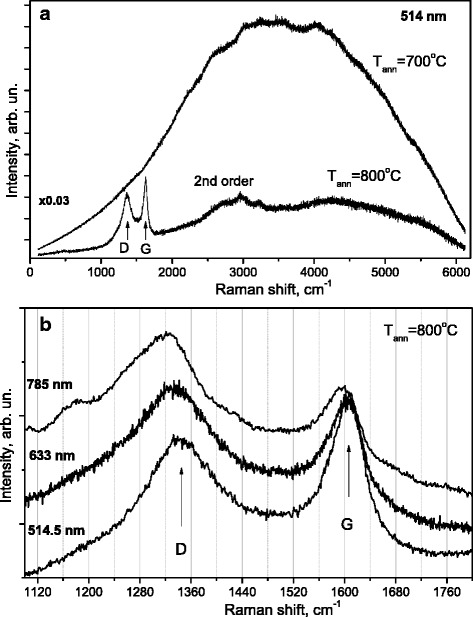
The comparison of Raman spectra measured in SiO_2_:C samples annealed at 700 and 800 °C with 514.5 nm excitation (**a**) along with the Raman spectra measured in SiO_2_:C sample annealed at 800 °C with different excitations (**b**)

As it is seen from Fig. 3a, the behavior of the SiO_2_:C sample annealed at 800 °C is essentially different from that for other samples—the PL appeared just as a very weak background and the D and G features typical of carbon were observed. The excitation with 514.5 nm enabled us to cover a broader spectral region; therefore, we recorded also the Raman spectrum of the SiO_2_:C sample annealed at 700 °C for the comparison. One can note the significant difference in the Raman spectra intensity measured in SiO_2_:C samples annealed at 700 and 800 °C.

In the SiO_2_:C sample annealed at 800 °C the D and G peaks are observed at the wavenumbers 1345 and 1607 cm^−1^, respectively, the triplet above 2720, 2946, and 3212 cm^−1^ are the Raman features corresponding to the second order scattering. As the exciting wavelength increases, the relative intensity of the D-band increases (with respect to the G-band) and at the same time, its frequency decreases (Fig. 3b). This behavior is typical of disordered carbons and of graphite as well [22–24]. There is also a shift of the G-band observed, but it is considerably smaller. It indicates that probably the powder is structurally very close to the nanocrystalline graphite.

### The Temperature Dependence of CW EPR Spectra in SiO_2_:C Nanopowders

**Fig. 4:**
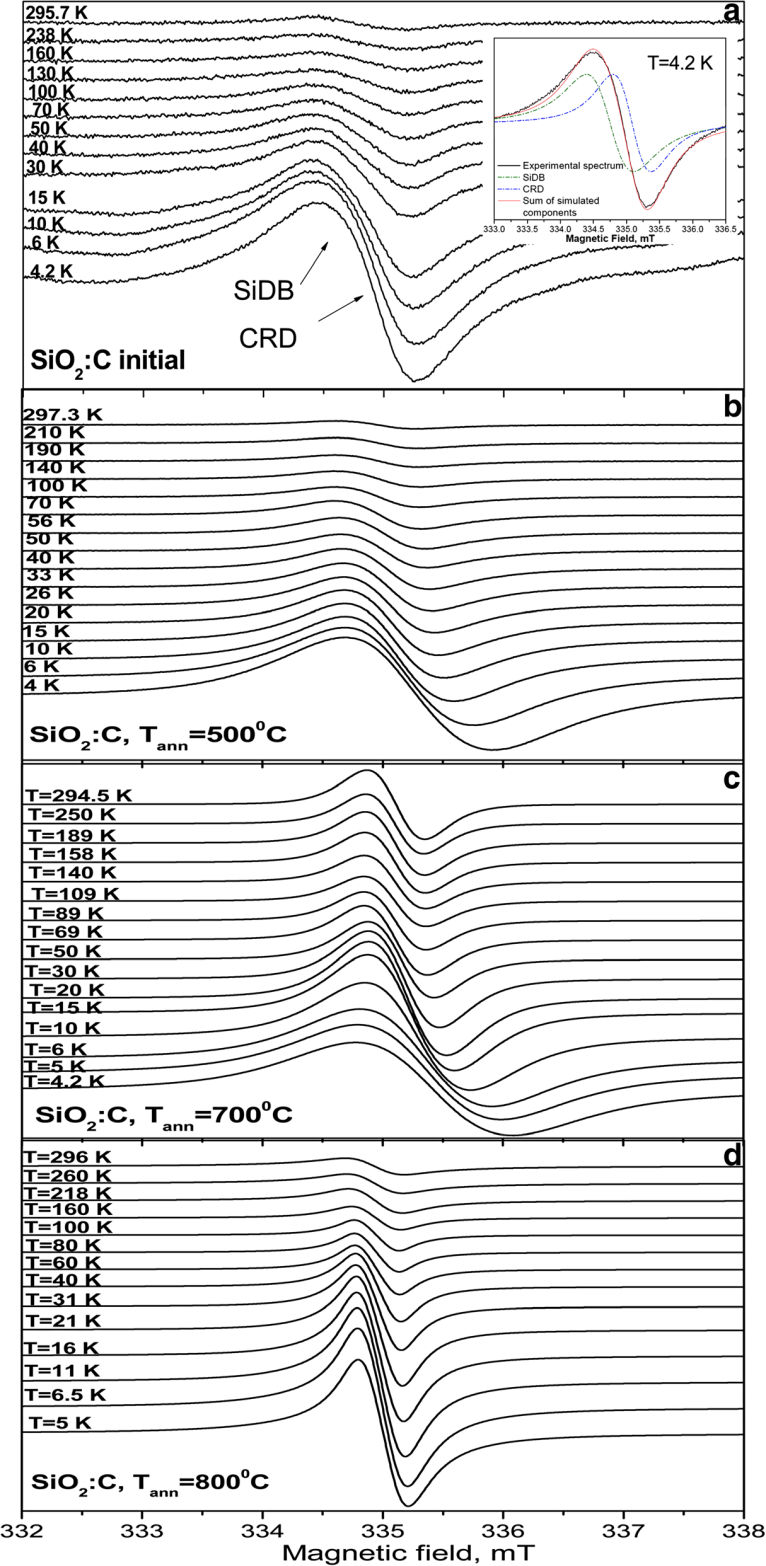
The temperature behavior of X-band CW EPR spectra measured in initial SiO_2_:C (**a**) and SiO_2_:C samples annealed at 500 °C (**b**), 700 °C (**c**), and 800 °C (**d**)

Figure 4 shows the temperature behavior of CW EPR spectra measured in initial SiO_2_:C samples and SiO_2_:C samples annealed at 500, 700, and 800 °C. In the initial SiO_2_:C sample two overlapping EPR signals of Lorentzian lineshape were observed in the whole temperature range. A weak EPR signal at *g* = 2.0055(3) was attributed to silicon dangling bonds SiDB [25], created on the surface of SiO_2_ particles after incomplete substitution of OH groups. The more intense signal with *g* = 2.0033(3) was assigned to carbon related defect (CRD). After the sample annealing, only one narrow Lorentzian EPR signal with temperature-dependent resonance magnetic field position, linewidth and signal intensity was observed in SiO_2_:C nanopowders. As can be seen from Table 1, the increase of annealing temperature leads to the increase of the spin concentration (*N*) of the observed carbon-related centers and its *g*-value.

**Table 1:** Parameters of EPR signals observed in the initial and annealed SiO_2_:C samples at 292 K

*T*_ann_	Paramagnetic center	*g*-factor	*N*, spins/cm^3^
Initial	SiDB	2.0055(3)	~1.5 · 10^17^
	CRD	2.0033(3)	~2.5 · 10^17^
500 °C	O-centered CRD	2.0042(3)	~3.3 · 10^18^
700 °C	C-centered CRD with a nearby O heteroatom	2.0035(3)	~4.1 · 10^22^
800 °C		2.0035(3)	~4.2 · 10^21^

**Fig. 5:**
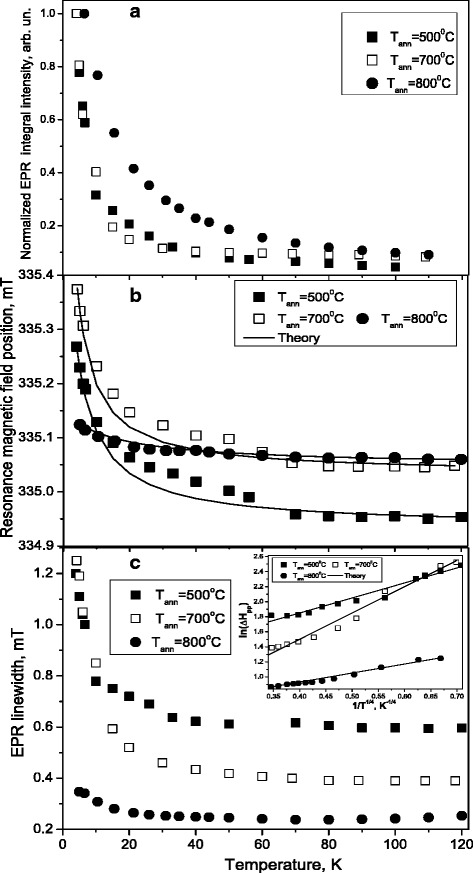
The temperature dependence of resonance magnetic field position (**a**), EPR signal integral intensity (**b**) and *EPR linewidth* (**c**) for CRR measured in SiO_2_:C samples annealed at 500, 700, and 800 °C

It is known that the carbon-centered radicals with an adjacent oxygen atom have *g*-factors in the range of 2.003–2.004, while oxygen-centered carbon radicals have *g* > 2.004 [26]. Therefore, we can suggest that the annealing of SiO_2_:C nanopowders at 500 °C leads to the formation of oxygen-centered radical, while the annealing at *T*_ann_ ≥ 600 °C leads to the formation of carbon-centered radicals with a nearby oxygen heteroatom [21]. This suggestion is also supported by the increase of the EPR signal linewidth. It was found that oxygen-centered and carbon-centered CRD have temperature dependent intensity, resonance field position and linewidth in the temperature range 120–4 K. Figure 5 shows the temperature dependence of resonance field position, normalized integral intensity and EPR linewidth for CRD measured in the temperature range from 6 to 120 K.

The temperature dependence of the integral intensity of the CRD EPR signal in the annealed SiO_2_:C samples, which is directly proportional to the EPR spin susceptibility (*χ*), has a tendency to increase with decreasing temperature from 100 to 4 K (see Fig. 5a) and can be described as:

1 $$ \chi (T)={\chi}_1+{\chi}_2=\frac{C}{T-\varTheta}+{\chi}_2, $$

where *χ*_1_ describes the Curie-Weiss law for the localized spins and *χ*_2_ is the temperature-independent contribution of the Pauli susceptibility of free electrons to the magnetic susceptibility of the localized spins due to the exchange interaction, *C* and Θ, the Curie-Weiss constant and temperature, correspondingly.

Figure 5b shows the temperature dependence of the resonance field position (*B*_*res*_) of the CRD EPR signal in the annealed SiO_2_:C samples measured in the temperature range from 120 to 5 K. The observed temperature variation of the *B*_*res*_ can be explained by the exchange interaction of the non-localized electrons of the CRD with the localized spin system, resulting in the change of a local magnetic field of CRD with temperature. Under strong exchange interaction, the overall behavior of the EPR signal is determined by one of the two spin systems and corresponds to the case when bottleneck regime is realized. As can be seen from Fig. 5b, at low temperatures, the localized spin system dominates the value of the *g*-factor of the EPR signal while conduction electron spins dominate its value at high temperatures.

The temperature dependence of resonance field for the narrow EPR line in all samples can be described by the exchange interaction between two different paramagnetic systems [27]:

2 $$ {B}_{res}(T)=\frac{{B_{res,}}_1{\chi}_1+{B_{res,}}_2{\chi}_2}{\chi_1+{\chi}_2}={B_{res,}}_2+\frac{\left({B_{res,}}_1-{B_{res,}}_2\right)\cdot \left({\chi}_1/{\chi}_2\right)}{\chi_1/{\chi}_2+1}, $$

where *B*_*res*,1_ and *B*_*res*,2_ are the resonance field positions for localized and non-localized spins, respectively.

The *g*-factors of the localized spin system, which were calculated using the following equation: *g*_1,2_ = *ω*/(*μ*_*B*_ *B*_*res*,1,2_), where *μ*_*B*_, the Bohr magneton and Θ, *C*/*χ*_2_ values extracted from the fitting of experimental data with theoretical description are presented in Table 2. The obtained small values of the negative/positive Curie-Weiss constants for the sample annealed at 500 and 700 °C suggest a weak antiferromagnetic/ferromagnetic interaction between magnetic moments of the localized spins. The Curie-Weiss contribution arises from the exchange interaction of the non-localized with a localized spin system, while Pauli-like contribution at *T* > 80 K serves as evidence of the metallic behavior of the CRD spin system.

**Table 2:** Parameters of non-localized (*g*_2_) and localized (*g*_1_) paramagnetic systems extracted from the fitting of Eq. (2) with experimental data in annealed SiO_2_:C samples

*T*_ann._	*g*_2_	*g*_1_	*C*/*χ*_2_	Θ, K
500 °C	2.0042(3)	2.0002(3)	3.4	−0.6
700 °C	2.0036(3)	1.9994(3)	2.6	1.5

The obtained *g*-values of 2.0002(3) and 1.9994(3) of the paramagnetic system with localized unpaired electrons are in a good agreement with those recently reported for the EPR signals from carbon nanodots, which could be an electron donor or electron acceptor [28, 29]. Carbon nanodots are spherical nanocrystals of graphite with a high concentration of oxygen due to their surface oxidation and their average size distribution is less than 10 nm. Therefore, we may assign the localized spin system with carbon nanodots having greater *sp* ^2^ character and contain lower amounts of carbon with higher oxygen contents, which also have been referred to as carbogenic nanodots in the literature [30, 31]. The presence of the oxygen in the nearest environment of the CRD in annealed SiO_2_:C nanopowders may be the reason for the formation of the carbogenic nanodots.

On the other hand, the ultrafine size of the dots combined with their disordered structure favors a high concentration of the surface defects. Therefore, the assignment of the paramagnetic system with localized unpaired electrons with carbon-containing interface E defect family with *g* ≈ 2.0005, which may form upon the thermal treatment, cannot be completely excluded as an alternative variant [32–34].

As it is seen from Fig. 5b, the *g*-factor of the CRD in the SiO_2_:C sample annealed at 800 °C does not significantly depend on the temperature. The weak temperature variation of the resonance field position for EPR line in SiO_2_:C powder annealed at 800 °C can be explained by a significant decrease of the spin concentration of the localized spin system.

As can be seen from Fig. 5c, the motional narrowing of CRD EPR signal linewidth Δ*H* was observed at very low temperatures from 4 to 20 K, which is not typical of nonmetallic materials and indicates that the electrons are mobile at 4 K. In this case, the hopping motion of the electron trapped in carbon nanodots can be described by variable-range hopping law, which is typical for disordered semiconductor structures [35]:

3 $$ \varDelta H(T)={\delta}_0 \exp \left({\left({T}_0/ T\right)}^{1/4}\right) $$

where *δ*_0_ is the EPR linewidth at *T* → ∝ due to spin-relaxation; *T*_0_ corresponds to the average barrier height for hopping motion and is described as: *T*_0_ = 16 *λ* ^−3^/*k*_*B*_ *N*(*E*_*F*_), where *λ* is the radius of the localized electron wave function, *N*(*E*_*F*_) is the density of states at the Fermi energy. From the fitting of the experimental data with Eq. (2) it, was found that *T*_0_ value in SiO_2_:C sample annealed at 500 °C is 1.38 meV, in the SiO_2_:C sample annealed at 700 °C it is equal to 12.9 meV and in the sample annealed at 800 °C it is 0.18 meV.

The mobility of the electrons at 4 K can be explained by the quantum character of the conductivity in the vicinity of the carbon layer [18]. The thermal excitation of the electrons cannot be the reason for this effect because the band gap for such small thickness of the C layer (of about 4 nm) becomes large enough. The fumed silica is an isolator and does not contain mobile electrons. Mobile electrons locate within the C layer, which has the typical size for C nanodots. This means that the electrons are trapped in a quantum well, but can move freely along the interface or the C layer surface [18].

Thus, the carbon nanodots can be considered as a quantum well for electrons. Electrons trapped in the quantum well can move from one carbon nanodot to another by hopping process through the energy barrier. The significant lowering of the energy barrier in the SiO_2_:C sample annealed at 800 °C may be explained by an increase of the radius of the localized electron wave function owing to the reconstruction of the nanostructured carbon layer (nanocrystalline graphite).

### ED EPR and Pulsed ENDOR Spectroscopy Study of SiO_2_:C Samples

It is well known that echo-detected EPR (ED EPR) method is capable to disentangle overlapping EPR lines by taking advantage of differences in spin lattice relaxation time of the paramagnetic centers. Another advantage of the echo experiment over CW technique is that the electron spin echo can be excited for inhomogeneously broadened EPR lines only. This allows immediate distinguishing between EPR signals from delocalized spins having homogeneously broadened EPR lines and the EPR signals from localized spins. Moreover, the hyperfine couplings responsible for the inhomogeneous broadening of the EPR lines, which cannot be directly observed in CW EPR experiments, can be resolved in the ED EPR spectrum.

**Fig. 6:**
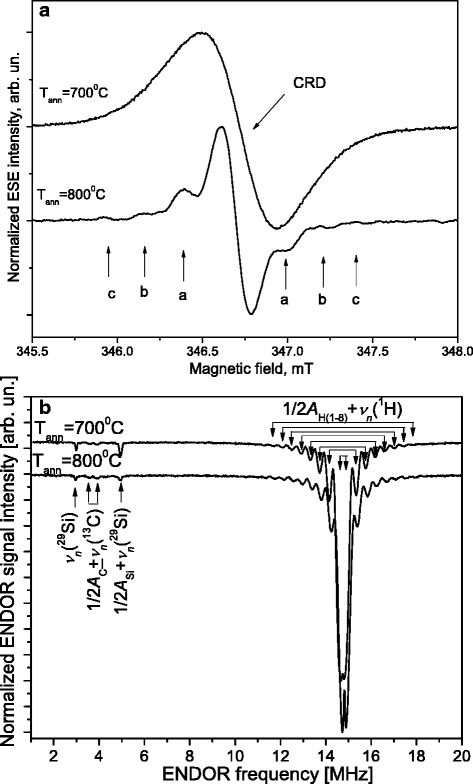
The first derivative of X-band ED EPR spectra measured in SiO_2_:C samples annealed at 700 °C (*upper traces*) and 800 °C (*lower traces*) (**a**). The X-band Mims pulsed ENDOR spectra measured in SiO_2_:C samples annealed at 700 °C (*upper trace*) and 800 °C (*lower trace*) when the magnetic field was set to the CRR field position. (**b**). *T* = 10 K

In the initial SiO_2_:C sample and SiO_2_:C samples annealed at *T*_ann_ ≤ 600 °C, no electron spin echo signal was detected. However, in the SiO_2_:C samples annealed at 700 and 800 °C we were able to record the ED EPR spectra (see Fig. 6a). Figure 6a shows the ED EPR spectra measured in SiO_2_:C samples annealed at 700 and 800 °C at *T* = 10 K. The *g*-factor of the ED EPR signals in both samples is *g* = 2.0035(3) that coincides with that found from EPR measurements for carbon-centered radical with a nearby oxygen heteroatom. We did not observe the shift of the resonance position for CRD line with the temperature decrease in ED EPR spectra as was observed in CW EPR spectra.

As can be seen from Fig. 6a, the ED EPR signal is broader in the SiO_2_:C sample annealed at 700 °C than that for the annealed at 800 °C. This can be explained by the shorter spin-lattice relaxation time for CRD in the SiO_2_:C sample annealed at 700 °C. Different relaxation times indicate that unpaired spins of CRD in SiO_2_:C sample annealed at 700 and 800 °C are in a different structural environment. The longer spin-lattice relaxation time for the CRD observed in SiO_2_:C sample annealed at 800 °C indicates that the local field dynamics in this sample is strongly suppressed.

The superhyperfine lines labeled as “a”, “b”, and “c” with the corresponding superhyperfine interaction constants of 0.46, 0.93 and 1.36 mT were resolved in the ED EPR spectra of SiO_2_:C sample annealed at 800 °C. From the intensity ratio of the satellite lines to the central line, we have concluded that they arise from the superhyperfine interaction of CRD with ^13^C and ^29^Si nuclei (both having a nuclear spin *I* = 1/2 and natural abundances of 1.1 and 4.7%, respectively).

The phase memory time (*T*_m_) for CRD in SiO_2_:C sample annealed at 800 °C was obtained from the time decay of the two-pulse electron spin echo signal amplitude, while the spin-lattice relaxation time (*T*_1_) was estimated from electron spin echo signal intensity changing under the variation of the shot repetition time. As a result, at *T* = 10 K for CRD we have obtained: *T*_m_ = 400 ns and *T*_1_ ≈ 1.6 μs.

We have measured the X-band Mims pulsed ENDOR spectra in SiO_2_:C samples annealed at 700 and 800 °C when the magnetic field was set to the CRD ED EPR line position (see Fig. 6b). The intense ENDOR signals originate from transitions 1/2*A*_i_ ± *ν*_n_(^1^H) (*ν*_n_(^1^H) – nuclear Larmor frequency of ^1^H) with superhyperfine interaction constants *A*_i_ ranging from 0.17 MHz to 6.16 MHz. The ENDOR spectra also revealed the weak signals from the transitions 1/2*A*_C_ ± *ν*_n_(^13^C) and 1/2*A*_Si_ + *ν*_n_(^29^Si) (*ν*_n_(^13^C), *ν*_n_(^29^Si) -nuclear Larmor frequency of ^13^C and ^29^Si) with superhyperfine interaction constants of *A*_C_ = 0.36 MHz and *A*_Si_ = 3.9 MHz, correspondingly. We suggest that after annealing at *T*_ann_ ≥ 700 °C the hydrogen from the broken methyl groups becomes available to saturate the carbon dangling bonds forming the (C-H) radicals. This suggestion is supported by the presence of carbon-hydrogen bonds (C(*sp* ^3^)-H) in the FTIR spectra of SiO_2_:C samples annealed at 700 and 800 °C.

**Fig. 7:**
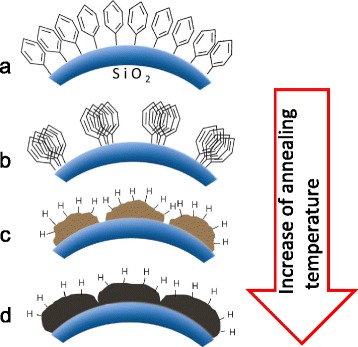
The structural modification of the carbon layer structure in SiO_2_:C with increase of the temperature annealing. **a** Initial SiO_2_:C. **b** *T*_ann_ = 500 °C. **c** *T*_ann_ = 700 °C. **d** *T*_ann_ = 800 °C

The presence of the hydrogen can be explained by thermal destruction of methoxy groups usually occurred with the formation of volatile products (CO, H_2_) at temperature below 500–600 °C. Partial decomposition of these groups results in formation of CH_n_ radicals that can be trapped by carbon dangling bonds in amorphous clusters/nanodots that are formed on the silica surface as a result of destruction of phenyl groups. Previously, it was reported [36] that C(*sp* ^3^)-H_n_ bonds can be stable in amorphous carbon-rich structures up to 850 °C. Figure 7 shows the structural modification of the carbon layer in SiO_2_:C with increase of the temperature annealing. Another reason for the presence of bonded hydrogen after high temperature annealing is chemisorption of organic contaminations and water molecules from atmospheric air by highly dispersive SiO_2_:C powder. From FTIR spectra it can be estimated that concentration of hydrogen chemically bonded with carbon is of several atomic percents that is sufficient to be detected in ENDOR spectrum of the CRD.

## Conclusions

Optical and magnetic properties of the initial and annealed in the temperature range of 500–800 °C carbonized silica (SiO_2_:C) nanopowders were studied by FTIR, Raman, CW EPR, and pulsed EPR methods, including ED EPR and pulsed ENDOR spectroscopy, in the temperature interval from 4.2 to 292 K. A strong rise of the PL intensity and shift of its maximum in the red range with the increase of the annealing temperature from 500 to 700 °C was observed in SiO_2_:C. The D and G bands that are typical of disordered carbons and graphite carbon were observed in Raman spectra of SiO_2_:C samples annealed at 800 °C when the intensity of the PL significantly decreases. The two overlapping EPR signals of Lorentzian lineshape were observed in the initial SiO_2_:C. The first EPR signal at *g* = 2.0055(3), which vanishes after thermal annealing, was assigned to silicon dangling bonds SiDB and the second one at *g* = 2.0033(3) is due to the carbon-related defect (CRD).

The effect of the thermal annealing on the EPR spectra of the SiO_2_:C nanopowders was studied in the temperature range of *T*_ann_ = 500–800 °C. We have found that the annealing treatment leads to the increase of the spin concentration and *g*-value of the CRD spin system. As a result the EPR signal at *g* = 2.0042(3) observed in SiO_2_:C annealed at 500 °C was attributed to the oxygen-centered CRD while EPR signal at *g* = 2.0035(3) observed in SiO_2_:C annealed at 700 and 800 °C was attributed to the CRD with a nearby oxygen heteroatom. The observed temperature-dependent variation of the resonance field position of the EPR line in annealed SiO_2_:C samples justifies that the resonance field of the CRD is governed by the exchange interaction of the non-localized electrons with localized electrons of the paramagnetic centers. The obtained *g*-values of 2.0002(3) and 1.9994(3) of the paramagnetic system with localized unpaired electrons are in a good agreement with those recently obtained for the EPR signals from carbon nanodots, which could be the electron donors or electron acceptor. Therefore, we may assign the localized spin system with carbogenic nanodots, which have greater *sp* ^2^ character and contain lower amounts of carbon with higher oxygen contents.

The weak temperature variation of the resonance field position of CRD EPR line in SiO_2_:C powder annealed at 800 °C can be explained by a significant decrease of the spin concentration of the localized spin system of carbon nanodots.

The temperature dependence of the integral intensity of the CRD EPR signal is described by the sum of the Curie-Weiss and Pauli paramagnetism. The observed motional narrowing of the EPR signal linewidth in the temperature interval from 4 to 20 K indicates that the electrons are mobile at 4 K and it can be explained by the quantum character of the conductivity in the vicinity of the carbon layer. Therefore, the carbon nanodots can be viewed as a quantum well for electrons. The electrons trapped in a quantum well can move from one carbon nanodot to another by variable-range hopping process through the energy barrier.

Pulsed EPR experiments have been used to distinguish between signals of delocalized and localized spins. The fact that the ED EPR signal at *g* = 2.0035(3) was detected only in the SiO_2_:C nanocomposites annealed at 700 and 800 °C justifies that the non-localized electrons coexist with localized electrons that have a superhyperfine interaction with surrounding ^13^C and ^29^Si nuclei located at the SiO_2_:C interface.

## Abbreviations

CRD: Carbon related defect
CW: Continuous wave
ED EPR: Echo-detected electron paramagnetic resonance
ENDOR: Electron nuclear double resonance
EPR: Electron paramagnetic resonance
FTIR: Fourier transform infrared spectroscopy
PL: Photoluminescence
PTMS: Phenyltrimethoxysilane
SiDB: Silicon dangling bonds
SiO_2_:C: Silica nanoparticles covered with carbon
